# VAT-Dependent Inflammatory Proteomic Signatures of Cardiometabolic Traits and Central Proteins

**DOI:** 10.21203/rs.3.rs-8411853/v1

**Published:** 2025-12-25

**Authors:** Azam Yazdani, Cong Wang, Olga Demler, Oluwafeyisola Osifala, Edward Giovannucci, Hong Zhang, Deirdre K. Tobias

**Affiliations:** 1. Division of Preventive Medicine, Department of Medicine, Brigham Woman’s Hospital, Boston, MA; 2. Harvard Medical School; 3. Swiss Federal Institute of Technology (ETH), Department of Computer Science, Zurich, Switzerland; 4. Harvard T.H.CHAN School of Public Health; 5. School of Nursing, Yale University; 6. Department of Nutrition, Harvard TH Chan School of Public Health, Boston, MA

**Keywords:** VAT-Dependent Markers, Inflammatory Proteomics, Cardiometabolic Traits, Visceral Adipose Tissue

## Abstract

Visceral adiposity tissue (VAT), fat located within the abdominal cavity, may play a causal role in driving inflammation and poor cardiometabolic health. This study investigates the cross-sectional relationships between multiple cardiometabolic traits, including VAT, and proteomic-based inflammatory signatures.

Body adiposity distribution quantified using dual-energy X-ray absorptiometry (DXA), cardiometabolic traits, and plasma proteomics inflammation panel (Olink Explore 384) were measured in a discovery cohort from the Vitamin D and Omega-3 Trial (VITAL; N = 525) and a replication cohort from the Cocoa Supplement and Multivitamin Outcomes Study (COSMOS; N = 371). We derived inflammatory proteomic markers of VAT, systolic blood pressure (SBP), high-density lipoprotein cholesterol (HDL-C), triglycerides (TG), fasting glucose, and insulin resistance (Homeostasis Model Assessment of Insulin Resistance; HOMA-IR). Inflammatory proteomic markers were identified via linear regression at a false discovery rate (FDR) < 0.05. Proteomic markers showed aligned associations with VAT and the other cardiometabolic traits, except for HDL-C, which was opposite. After adjusting for VAT levels, most proteomic markers were no longer statistically significant: >97% for glucose and SBP, and 73%, 62%, and 56% for HOMA-IR, TG, and HDL-C, respectively, suggesting that VAT explains the variability of these associations. To further elucidate shared mechanisms, we examined the network architecture of 86 proteomic markers common to all cardiometabolic traits. Some of the VAT-dependent protein signatures with high centrality were TGFB1, PDLIM7, COLEC12, and LAIR1. These hub-like proteins may reflect the influence of VAT accumulation on other cardiometabolic traits and highlight novel therapeutic targets for reducing cardiometabolic risk.

## Introduction

Growing evidence suggests that visceral adiposity, fat located within the abdominal cavity, plays a direct role in driving inflammation and cardiometabolic dysfunction^1–3^. Despite visceral adiposity being an important risk factor for cardiometabolic diseases and cancer, the mechanisms through which visceral adiposity contributes to the development of complex chronic diseases remain unclear. Current evidence on visceral adiposity and inflammation has been limited to general inflammatory biomarkers, such as C-reactive protein, and selected cytokines, such as interleukin-6, without a comprehensive evaluation using inflammation-focused proteomics. We sought to derive proteomic-based inflammation signatures of visceral adipose tissue (VAT), quantified using dual-energy X-ray absorptiometry (DXA), as well as VAT-related cardiometabolic traits. Specifically, we analyzed VAT mass, gynoid region fat mass (GYN), truncal fat mass (TRUNK), all of them in grams, and whole-body fat mass (WBF) in kilograms. We examined the overlap between inflammatory proteins associated with VAT and other cardiometabolic traits, including systolic blood pressure (SBP), high-density lipoprotein cholesterol (HDL-C), low-density lipoprotein cholesterol (LDL-C), triglycerides (TG), fasting glucose, and insulin resistance (Homeostasis Model Assessment of Insulin Resistance; HOMA-IR).

The discovery cohort for deriving inflammatory proteomic markers included general population adults without complex diseases from the US-based Vitamin D and Omega-3 Trial (VITAL, N = 525)^4^. Our replication cohort is from the Cocoa Supplement and Multivitamin Outcomes Study (COSMOS, N = 371)^5^. We evaluated the overlap of protein markers between VAT and other cardiometabolic traits and examined whether VAT may serve as a potential underlying driver of the proteomic–cardiometabolic trait associations. Finally, for mechanistic insights into proteomic inflammation of VAT and other cardiometabolic traits, we investigated the network architecture of proteomic signatures shared among these cardiometabolic traits and identified proteins with high centrality that may serve as proxies for broader inflammatory or metabolic activity within the network. The VAT-dependent hub-like proteins could reflect the influence of VAT on other cardiometabolic traits and may highlight novel therapeutic targets for reducing cardiometabolic risk.

## Methods

### Data Availability.

The VITAL study measured the cardiometabolic traits and DXA measurements in a sub-cohort at the Clinical Translational Science Center at baseline. Requests to access the dataset from qualified researchers trained in human subject confidentiality protocols should be sent to the Steering Committees of the parent trial.

### Cohorts.

VITAL was a double-blind, placebo-controlled randomized trial initiated in 2011 to evaluate the effects of vitamin D and marine omega-3 fatty acid supplementation for the primary prevention of cancer and cardiovascular disease (NCT01169259)^4^. COSMOS was a double-blind, placebo-controlled trial to evaluate the effects of cocoa extract and multivitamin supplements for the prevention of cancer and cardiovascular disease (NCT02422745)^5^. For both cohorts, eligible Boston-area participants without cancer or cardiovascular disease attended baseline in-person clinic visits for in-depth phenotyping, DXA whole body scans, and biospecimen collections.

### DXA.

Visceral adiposity was estimated using full-body DXA scans, a low-radiation alternative to computed tomography (CT) that provides comparable quantification^6^. Clinical fat depots were measured in a 5 cm abdominal region above the iliac crest, corresponding to the CT measurement area. The software automatically identifies the outer and inner abdominal wall margins at the level of the fourth lumbar vertebra and measures total fat mass within this region, which includes both subcutaneous and visceral fat. Subcutaneous fat above and below the visceral region is then estimated and subtracted from the total abdominal fat to derive VAT mass.

### Cardiometabolic traits.

The traits HDL-C, LDL-C, glucose, and triglycerides were measured from fasting blood samples during in-clinic assessments. Oral glucose tolerance testing was performed, and the HOMA-IR index was calculated from fasting insulin and glucose values. SBP was assessed using direct blood pressure measurement during physical examination^7^.

### Proteomics measurement.

All plasma samples were assayed with the Olink Explore 384 Platform (https://olink.com/products/olink-explore-3072-384, Olink, Watertown, MA). This high-throughput platform measures 384 inflammation-related proteins using a multiplexed proximity extension assay (PEA). Protein pairs are targeted by antibodies conjugated to complementary oligonucleotides, which hybridize upon binding and enable amplification for relative quantification via next-generation sequencing. Olink includes internal controls for incubation, extension, and amplification, as well as external negative, plate, and sample controls. Limits of detection (LOD) are calculated per plate using triplicate negative controls. Normalized protein expression (NPX) values are derived through extension control normalization, log2 transformation, and plate control adjustment. Samples and assays are flagged if quality metrics deviate from predefined thresholds.

### Proteomics quality control.

For the quality control of proteomics, we took the steps below: 1) Removing samples with QC warnings, 2) Removing proteins with records below the protein-specific limit of detection (LOD) in more than 50% of observations. 3) For assays with values below LOD, the actual value was used, 4) Winzorizing proteins to 5% to 95% to limit the impact of outliers while preserving the overall distribution of the data, and 5) Standardization (mean = 0 and standard deviation = 1) to have the effect size comparable across proteins.

## Analytical Approach

### Linear regression.

We first performed linear regression analyses, fitting each of the cardiometabolic traits and each of the DXA measurements to each of the proteins. Data preprocessing for cardiometabolic traits and DXA measurements included normalization by log-transformation, winsorization to 5^th^ and 95^th^ percentiles, and standardization (mean = 0 and standard deviation = 1). All models were adjusted for the set of covariates age, sex, race/ethnicity, and batch effect. We selected the significant associations using the Benjamini-Hochberg method, false discovery rate (FDR) < 0.05. The results of this analysis are provided in Figure 2 for cardiometabolic traits and Table 2 for DXA measurements.

In a secondary analysis, we adjusted models for aspirin and another time for statin use in addition to the set of covariates.

To identify VAT-dependent protein markers of cardiometabolic traits, we adjusted the regression models for VAT in addition to the set of covariates.

### Network architecture.

For mechanistic insights into proteomic inflammation of VAT and other cardiometabolic traits, we investigated the network architecture of proteomic signatures shared among these cardiometabolic traits. To infer the network architecture, we used data-driven networks, where proteins are represented as nodes, and the edges represent direct connections that cannot be fully explained by any subset of other proteins^8–10^. These networks offer a view of the underlying inflammatory proteomic network architecture. Further network analysis identified proteins with high centrality that may serve as proxies for broader inflammatory or metabolic activity within the network.

We first normalized and then adjusted the proteins for batch effects, sex, age, and race/ethnicity. We then identified a Bayesian network^11^ at level alpha = 1e-3 selected using SPOT^12^, where the Hamming distance of different networks is modeled on two consequence levels of alpha,

$$\alpha_{i}=\left\{10^{-i}|i=2,3,4,5\right\},$$

where *α*_*i*_ represents the significance level used in statistical tests to determine an edge between two molecules, conditioning on all sets of other molecules in the study and identified the optimal *α*_*i*_.

## Results

**Figure 1.**
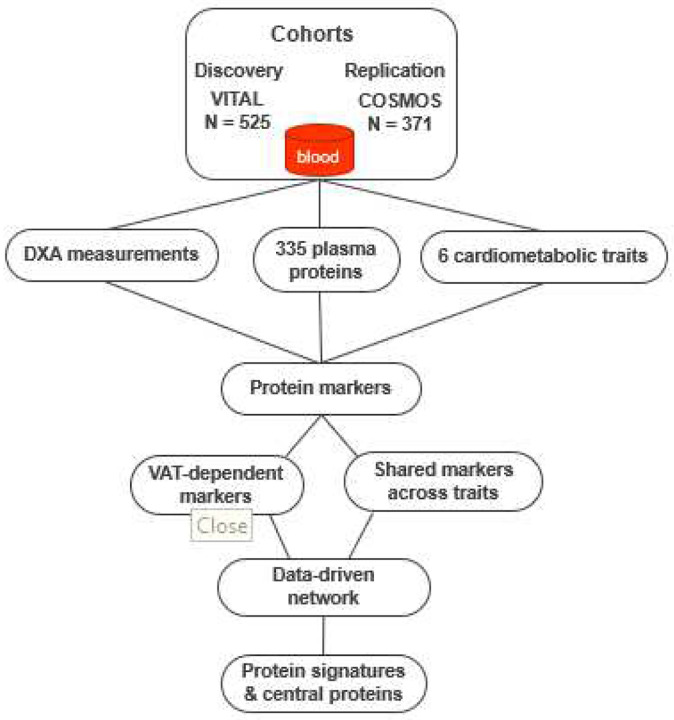
Overview of the study. We identified protein markers associated with six cardiometabolic traits as well as DXA measurements of fat. Shared protein markers across traits and VAT-dependent protein markers of cardiometabolic traits were analyzed with data-driven networks to identify protein signatures and central proteins.

Our analyses included N = 525 eligible participants from the VITAL baseline dataset as the discovery cohort and 371 participants from the COSMOS baseline dataset as the replication cohort. The workflow of this study is provided in Figure 1. The baseline characteristics of the discovery and replication cohorts are summarized in Table 1. Both cohorts have similar BMI and approximately 50% female participants. However, participants in the replication cohort are on average 5 years older and have a 10% higher proportion of White compared to the discovery cohort.

### Inflammatory protein markers of DXA measurements.

We identified 221 VAT signatures at FDR < 0.05 (66% of tested inflammatory proteins), with the most markers among other DXA fat mass measurements, including GYN, TRUNK, and WBF. The markers of VAT included the majority of GYN, TRUNK, and WBF markers (>96%) as well as an additional 53 markers unique to VAT, and VAT markers had the highest replication rate in COSMOS among other DXA measurements (Table 2).

### Protein markers of cardiometabolic traits and their overlap with VAT.

**Figure 2.**
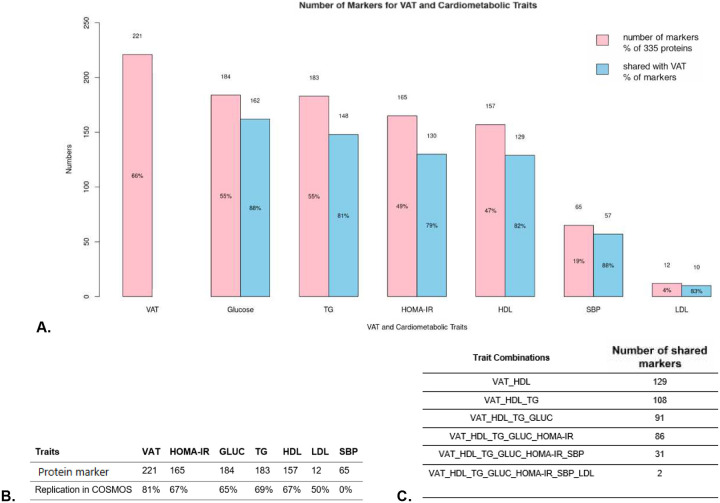

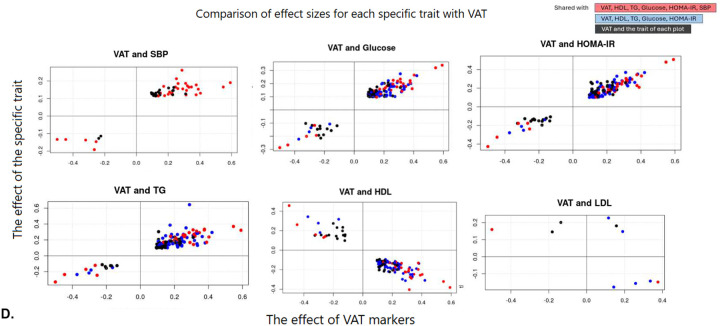
Overlap of VAT markers with cardiometabolic traits. **A.** The number of protein markers for each trait (pink bars) and their overlap with VAT (blue bars). **B.** Replication of VAT and cardiometabolic trait markers. **C.** VAT markers shared across traits: 86 signatures shared among HDL-C, TG, glucose, and HOMA-IR. **D.** Comparison of effect sizes: markers shared across all traits showed the largest effect sizes (red dots). The sign of effects were consistent between VAT and TG, glucose, HOMA-IR, and SBP; whereas HDL-C effects were reversed, and LDL-C showed mixed signs.

We identified protein markers associated with cardiometabolic traits at FDR < 0.05, up to 184 markers for glucose (55% of tested proteins), 183 for TG (55%), 165 for HOMA-IR (49%), 157 for HDL-C (47%), 65 for SBP (19%), and 12 for LDL-C (4%), these results are depicted in (Figure 2A), and the replication in COSMOS is provided in (Figure 2B). A total of 86 (39%) VAT-associated markers were shared across HDL-C, TG, glucose, and HOMA-IR, and 31 of these (14%) also overlapped with SBP. Due to the limited number of LDL-C markers, only 2 VAT markers (0.9%) were shared across all cardiometabolic traits (Figure 2C).

All VAT signatures shared with HOMA-IR, glucose, TG, or SBP followed the same direction, whereas those shared with HDL-C were inverse (Figure 2D). These findings were replicated in COSMOS.

Adjusting the models for aspirin use at the time of baseline visits, the number of proteins significantly associated with SBP was reduced from 65 to 49, but there were minimal changes among the other traits or VAT. Adjusting the models for statin use, the number of proteins significantly associated with LDL-C was reduced from 12 to 3, but the change in the protein markers of other traits was minimal, (see supplementary files).

### Network architecture of shared signatures between VAT and the other cardiometabolic traits.

**Figure 3.**
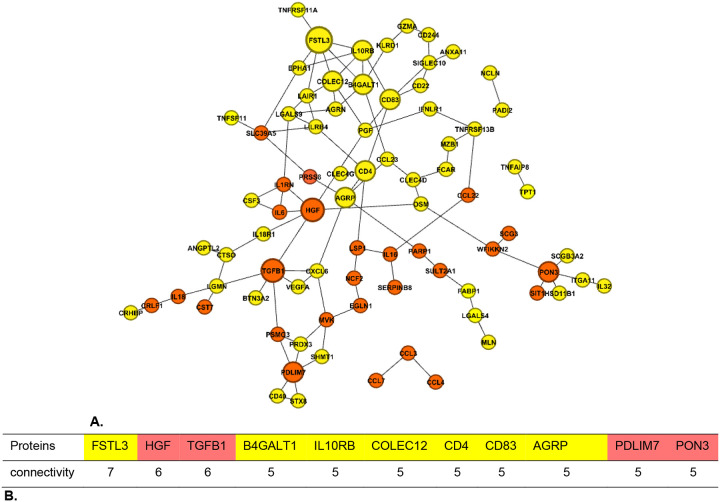
The proteomic connectivity underlying cardiometabolic traits shared with VAT. **A.** The data-driven network of 86 VAT signatures shared across HDL, TG, glucose, and HOMA-IR. Proteins also shared with SBP are highlighted in orange. Edges denote direct connectivity. Node size reflects the number of connectivity, with larger nodes indicating higher centrality. A yellow module at the top was identified with no markers shared with SBP, and an orange pathway-like pattern at the bottom was identified as being shared across all traits, including SBP. Dense modules, such as the yellow one at the top, suggest stronger inter-protein connectivity, whereas sparser pathways, such as the orange one, indicate more independent behavior. **B.** Connectivity representing the centrality of the specific protein. The number of connections indicates how many proteins a specific protein is directly linked to, and here we provide a list of protein markers shared across traits with high connectivity, representing the centrality of each protein.

We identified a data-driven network of 86 VAT signatures shared with HDL-C, TG, glucose, and HOMA-IR, and revealed their direct and indirect connectivity (Figure 3A). On this network, we mapped the subset of 31 signatures that were additionally shared with SBP. We also extracted proteins with the highest connectivity in the network (Figure 3B). These proteins may capture VAT-associated inflammatory processes that are linked to cardiometabolic traits.

There is a set of signatures (located at the top of the network) that is not shared with SBP and includes highly connected signatures, such as FSTL3, B4GALT1, IL10RB, COLEC12, CD4, CD83, and AGRP. This set may highlight mechanisms underlying VAT associations with HDL, TG, glucose, and HOMA-IR that are not shared with SBP. The 31 signatures shared across traits, including SBP, comprise highly connected proteins such as HGF, TGFB1, PDLIM7, and PON3 (Figure 3B).

### VAT-dependent protein markers of cardiometabolic traits.

**Figure 4.**
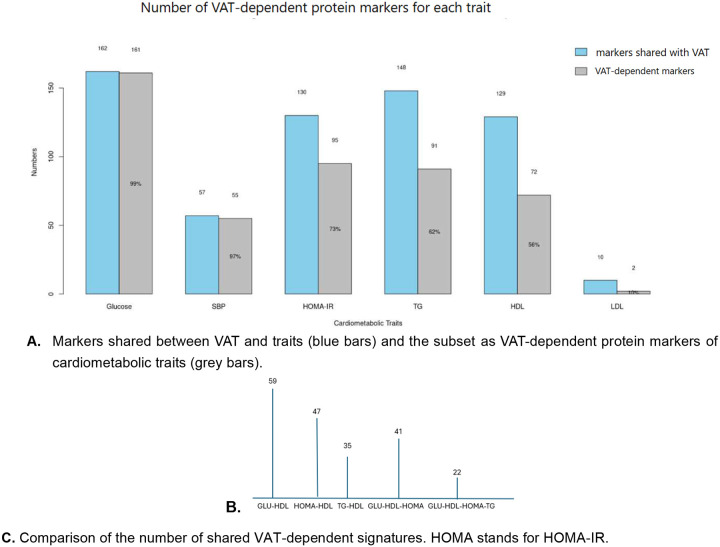
VAT-dependent signatures of cardiometabolic traits. **A.** Protein markers shared between VAT and traits (blue bars) and those losing associations after VAT adjustment (grey bars). **B.** Number of VAT-dependent markers shared across traits; HDL shares 35 VAT-dependent proteins with TG, fewer than the 41 shared among HDL, Glucose, and HOMA-IR combined.

Among protein-trait associations shared with VAT (Figure 4A, blue bars), those that lost significance (FDR > 0.05) after adjusting for VAT represent VAT-dependent markers of cardiometabolic traits, (Figure 4A, grey bars), up to 161 VAT-dependent markers for glucose (99% of shared markers with VAT), 55 markers for SBP (97%), 95 markers for HOMA-IR (73%), 91 markers for TG (62%), 72 markers for HDL (56%), and 2 markers for LDL-C (10%).

### Network architecture of VAT-dependent signatures in cardiometabolic traits.

**Figure 5.**
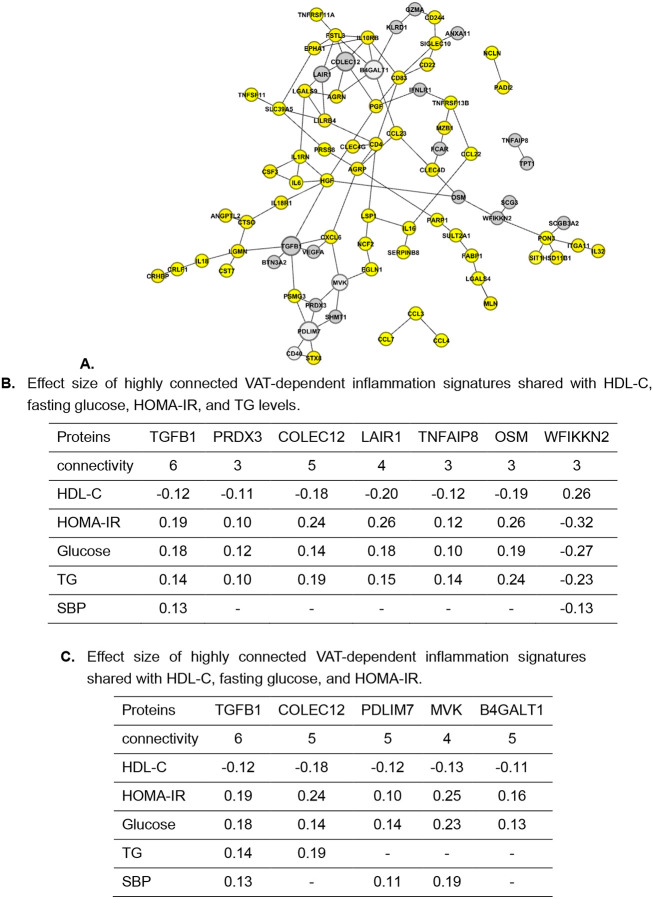
Network architecture of VAT-dependent signatures shared across HDL-C, Glucose, HOMA-IR, and TG. **A.** VAT-dependent signatures (the greys) mapped on the network of 86 VAT signatures shared across cardiometabolic traits; the VAT-dependent signatures in light grey were not shared with TG, including highly connected proteins B4GALT1, PDLIM7, and MVK. This means these proteins did not lose their association with TG after adjusting for VAT. **B.** Highly connected VAT-dependent signatures shared with all four traits: HDL-C, Glucose, HOMA-IR, and TG. The connectivity of each protein indicates the number of other proteins to which it is directly connected. **C.** Highly connected VAT-dependent signatures shared among HDL-C, HOMA-IR, and Glucose.

We mapped the VAT-dependent signatures onto the network of 86 signatures of cardiometabolic traits shared with VAT (Figure 5A). The dark grey nodes represent VAT-dependent signatures shared with glucose, HOMA-IR, HDL-C, and TG, whereas the light-grey nodes are not shared with TG. Among VAT-dependent signatures that are not shared with TG, some of the highly connected proteins include B4GALT1, PDLIM7, and MVK.

The highly connected VAT-dependent signatures of the cardiometabolic traits are represented in Figure 5B. While VAT-dependent signatures were mostly connected in pairs, the highly connected signatures TGFB1 and PDLIM7 formed a module (Figure 5B, bottom of network), suggesting the proteins in this module may share related underlying mechanisms.

## Discussion

Our findings support the hypothesis that visceral adiposity plays a key role in modulating inflammatory processes related to cardiometabolic traits. Using proteomics and powerful analytic approaches, we characterized inflammatory protein signatures associated with VAT and found them to be abundant. This supports the notion that VAT is a major contributor to multiple pathways of systemic inflammation and cardiometabolic dysfunction. The greater number of associations between inflammatory proteins and VAT compared with other cardiometabolic markers may reflect a more direct role of VAT in initiating and sustaining inflammation, whereas the other traits are complex phenotypic outcomes influenced by multiple biological systems. These findings also emphasize the importance of investigating the role of adiposity composition and regional location as unique contributors to poor cardiometabolic outcomes.

One of the most striking findings is the extensive overlap between VAT proteomic inflammation signatures and the signatures of the other cardiometabolic traits that we evaluated, including glycemic control, blood lipids, and blood pressure. Over 80% of inflammatory protein markers identified for fasting glucose, TG, HDL-C, and HOMA-IR were also significantly associated with VAT, suggesting a shared underlying inflammatory phenotype. Importantly, a subset of these shared proteins was no longer associated with the individual cardiometabolic traits when models were adjusted for VAT, indicating that VAT likely mediates or explains the link between inflammation and other measures of cardiometabolic health. VAT accounted for over 97% of the inflammation markers for SBP and fasting glucose, and a majority for HOMA-IR and TG, underscoring its central role in this phenotypic network. The directions of associations of the shared inflammation signatures were similar between VAT and HOMA-IR, glucose, TG, and SBP, whereas the signatures shared by VAT and HDL-C were in the opposite direction, as expected given by the inverse association of HDL-C with cardiometabolic risk.

To further investigate the interplay between VAT and inflammation and other cardiometabolic traits, we used a data-driven network to identify the network architecture of shared inflammatory protein signatures across these traits. Among 86 VAT inflammation markers shared across cardiometabolic traits, the proteins FSTL3, HGF, TGFB1, B4GALT1, IL10RB, COLEC12, CD4, CD83, AGRP, PDLIM7, and PON3 had the highest connectivity, each being directly connected to 5–7 other proteins, indicating their central role within the network. Among VAT-dependent markers of cardiometabolic traits, the proteins TGFB1, HGF, PDLIM7, and COLEC12 had the highest connectivity. These proteins may serve as proxies for VAT-driven inflammatory pathways influencing cardiometabolic risk, highlighting potential targets for mechanistic studies or therapeutic intervention. The highly connected proteins B4GALT1, PDLIM7, and MVK in the network were VAT-dependent signatures of glucose, HDL, and HOMA-IR but not TG, which means that after adjusting for VAT, these proteins remain significantly associated with TG. This suggests that their relationship with TG reflects a biological pathway linking protein regulation directly to triglyceride metabolism.

This study leverages objective DXA-based measurements of visceral adiposity and comprehensive Olink proteomics in two well-phenotyped, population-based cohorts with a discovery–replication design. The analysis integrates multiple cardiometabolic traits using rigorous statistical methods and demonstrates that VAT explains much of the inflammatory proteomic variation seen in other traits. The use of the data-driven network provided novel mechanistic insight and identified central, VAT-dependent hub proteins that may represent therapeutic targets. This study focused exclusively on an inflammatory panel of 335 candidate proteins, which, while informative, omits proteins from other biological pathways (e.g., immune function) that may also be relevant to the interplay of visceral adiposity and cardiometabolic risk. Since body compositions, inflammatory proteomics, and cardiometabolic traits were measured at the same time, the cross-sectional analyses limit the interpretation of the temporality among VAT, inflammation, and other cardiometabolic traits; however, the rich data collection and phenotyping of the clinical cohorts serves as a significant strength, allowing us to estimate the association of VAT with the proteomic and cardiometabolic trait networks independent of other risk factors and confounders. The sample sizes were also moderate and primarily composed of older adults, potentially limiting the generalizability of the findings to younger populations. Additionally, while DXA provides robust estimates of fat distribution, it does not capture functional or histological aspects of adipose tissue that may also influence inflammation.

In conclusion, this study provides evidence that VAT is a central feature of inflammatory processes underlying cardiometabolic abnormalities. Our findings offer new insights into the molecular pathways connecting fat distribution and metabolic dysfunction and highlight potential protein targets that could guide prevention or therapeutic strategies in individuals with excess visceral fat.

## Figures and Tables

**Table 1.** Baseline characteristics of participants in VITAL (the discovery cohort) and COSMOS (the replication cohort)

Characteristics	VITAL (n = 525)	COSMOS (n = 371)
Age (mean)	64±6.1	69±5.2
Female%	49	52
Body mass index (BMI, kg/m2)	28±4.8	28±5.3
Race%	86% White	96% White
Aspirin use	224 (42.6%)	
Statin use	154(29.3%)	
**Anthropometric measures by DXA, Median [Q1, Q3**]		
VAT (g)	741 [512,969]	739 [483,1019]
TRUNK	14,196 [10,579,17962]	14,367[10,495,18,633]
GYN	4,293 [3,517, 5,495]	4,433 [3,605, 5,629]
WBF	27 [22, 34]	27 [21, 35]
**Cardiometabolic traits, Median [Q1, Q3]**		
Fasting glucose (mg/dL)	97 [92, 103]	98 [92, 104]
HOMA-IR	1.8 [1.1, 2.7]	1.9 [1.1, 2.9]
HDL-C	51 [42, 64]	60[49,74]
LDL-C	114 [96,133]	116 [95.5,136.0]
TG	90 [67, 122]	86 [65,123]
SBP	122 [112,130]	130 [119, 140]

**Table 2.** Comparison of DXA fat mass compartment measurements based on inflammatory protein markers

DXA measurements	WBF	TRUNK	GYN	VAT	BMI
Protein markers	143	171	102	221	142
Shared markers with VAT	140	168	98		140
Replication in COSMOS	78%	80%	76%	81%	78%
